# Polyhydroxyalkanoate production from animal by‐products: Development of a pneumatic feeding system for solid fat/protein‐emulsions

**DOI:** 10.1111/1751-7915.14150

**Published:** 2022-09-27

**Authors:** Björn Gutschmann, Thomas H. Högl, Boyang Huang, Matilde Maldonado Simões, Stefan Junne, Peter Neubauer, Thomas Grimm, Sebastian L. Riedel

**Affiliations:** ^1^ Technische Universität Berlin, Chair of Bioprocess Engineering Berlin Germany; ^2^ ANiMOX GmbH Berlin Germany

## Abstract

Fat‐containing animal by‐product streams are locally available in large quantities. Depending on their quality, they can be inexpensive substrates for biotechnological processes. To accelerate industrial polyhydroxyalkanoate (PHA) bioplastic production, the development of efficient bioprocesses that are based on animal by‐product streams is a promising approach to reduce overall production costs. However, the solid nature of animal by‐product streams requires a tailor‐made process development. In this study, a fat/protein‐emulsion (FPE), which is a by‐product stream from industrial‐scale pharmaceutical heparin production and of which several hundred tons are available annually, was evaluated for PHA production with *Ralstonia eutropha*. The FPE was used as the sole source of carbon and nitrogen in shake flask and bioreactor cultivations. A tailored pneumatic feeding system was built for laboratory bioreactors to facilitate fed‐batch cultivations with the solid FPE. The process yielded up to 51 g L^−1^ cell dry weight containing 71 wt% PHA with a space–time yield of 0.6 g_PHA_ L^−1^ h^−1^ without using any carbon or nitrogen sources other than FPE. The presented approach highlights the potential of animal by‐product stream valorization into PHA and contributes to a transition towards a circular bioeconomy.

## Graphical abstract

In this study, a fat/protein‐emulsion (FPE), which is a by‐product stream from industrial‐scale pharmaceutical heparin, was evaluated for PHA production with *Ralstonia eutropha*. A tailored pneumatic feeding system was built for laboratory bioreactors to facilitate fed‐batch cultivations with the solid FPE. The developed process yielded up to 51 g L^−1^ cell dry weight containing 71 wt% PHA with a space–time yield of 0.6 g_PHA_ L^−1^ h^−1^ without using any carbon or nitrogen sources other than FPE.

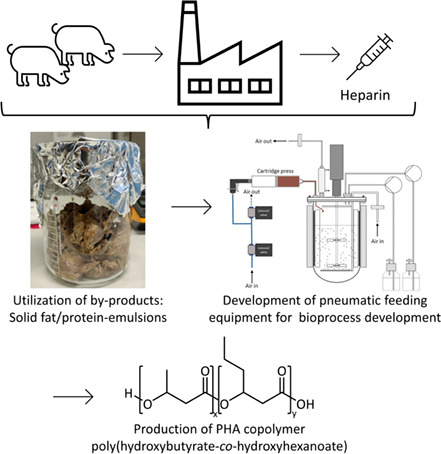

## INTRODUCTION

To accomplish a transition from fossil to renewable carbon‐based processes, the production of plastic from alternative feedstocks must be established. Drop‐in solutions that are based on renewable or residual resources can help to replace traditional fossil plastic with identical bio‐counterparts (Rosenboom et al., 2022). However, the biodegradation feature of bioplastics needs to be critically assessed in order to protect the environment from the exposure of microparticles that last within ecosystems. Environmental factors such as pH, temperature or oxygen availability influence the degradation behaviour (Ferreira & Åkesson, 2020). A corresponding label is awarded by the TÜV Austria (Vienna, Austria), to prevent the greenwashing of products with a simple “biodegradable” label. Polyhydroxyalkanoate (PHA) are bio‐based polymers and they are degradable in all of the test environments: soil, industrial and home composts, seawater and freshwater (Ferreira & Åkesson, 2020; Laycock et al., 2020).

PHAs are a group of linear polyesters, which are synthesized by various microorganisms as energy and carbon storage compounds. The current trend in literature focuses on the conversion of biogenic by‐products or waste streams to PHA to establish an inexpensive production, which thus competes with low‐cost conventional plastic (Riedel & Brigham, 2020). Such valorization is a great opportunity to accelerate a transition towards a circular bioeconomy. In particular, oleaginous residuals represent a very promising feedstock due to the high carbon content, low culture dilutions in fed‐batch processes, high conversion rates to PHA and the possibility to produce short‐chain length (*scl*, C‐atoms <6) and medium‐chain length (*mcl*, 5 < C‐atoms < 15) PHA or copolymers thereof (Lakshmanan et al., 2020; Riedel et al., 2014).

Animal by‐product streams from the animal processing industry are a very abundant and locally available feedstock (Gutschmann, Huang, et al., 2022; Koller et al., 2018). Depending on their quality, animal by‐product streams can be distinguished into three categories (European Council, 2009): high‐risk material, which has to be incinerated (Category 1); medium‐risk material, which can be additionally used for biogas and fertilizer production (Category 2); and low‐risk material, which has the least restrictions in their range of application (Category 3). Animal by‐product streams contain a large fraction of fats and proteins, which can be converted to high‐value products. Typically, animal by‐product streams are processed via thermal pressure treatments to separate fat and protein fractions (Toldrá et al., 2021). The proteins are used as functional ingredients, protein hydrolysates (as bioactive peptides, taste enhancers, pet food and feed), bioplastics, pharmaceutical applications, coagulants and flocculants for waste‐water treatment or wood adhesives. Animal‐derived waste fats are used in oleochemical industry for the production of cosmetics, feeds, soaps, detergents, pharmaceuticals, bioplastic or biodiesel (Toldrá et al., 2021).

Conversion of waste animal fats and mixed fat fractions to PHA has been demonstrated in multiple studies at the shake flask scale (Acedos et al., 2022; Ashby & Foglia, 1998; Cromwick et al., 1996; Riedel et al., 2015; Rodríguez et al., 2021; Saad et al., 2021; Taniguchi et al., 2003). A subsequent process transfer to the bioreactor scale is challenging due to the hydrophobicity and high melting temperature of waste animal fats. One approach to tackle this problem is the preliminary conversion of waste animal fats to biodiesel and the subsequent usage of the glycerol and saturated fatty acid methyl ester phases for PHA production (Koller & Braunegg, 2015; Muhr et al., 2013). Another strategy is the thermal liquefaction of waste animal fats and direct feeding into an existing emulsion formed with plant oil in *Ralstonia eutropha* cultivations (Gutschmann, Maldonado Simões, et al., 2022; Riedel et al., 2015). The latter approach makes use of a natural emulsification process, which is catalysed by extracellular lipases and eventually stabilized by extracellular polysaccharides (Gutschmann et al., 2021; Lu et al., 2013).

The pharmaceutical production of the anticoagulant agent heparin is a process in which the product is derived from porcine intestinal mucosa (FDA, 2013). The manufacture of heparin involves the extraction and isolation of crude heparin from porcine intestinal mucosa and the further purification of heparin. Heparin appears at a low concentration in the starting material (160–260 mg kg^−1^) (Van Der Meer et al., 2017). Therefore, large amounts of by‐products can be generated during the extraction and purification process. Fat/protein‐emulsions (FPEs) are one of the by‐product streams. A world leader in the production of heparin as an active pharmaceutical ingredient (API) is BIOIBERICA S.A.U.^1^ In different production plants of BIOIBERICA, hundreds of tons of FPE are generated each year, which makes it an interesting feedstock for high‐value applications. In a previous study, such an FPE was identified as a potential feedstock for PHA production with *R*. *eutropha* in a shake flask screening (Saad et al., 2021).

This current study describes the possibility to use FPE as the sole carbon and nitrogen source in a shake flask screening and evaluates the feedstock in laboratory bioreactor cultivations. For this purpose, a novel pneumatic feeding system was developed to feed the solid FPE with a tailored profile into the bioreactor and produce PHA at high cell densities.

## EXPERIMENTAL PROCEDURES

### Bacterial strain and growth media

The engineered *R. eutropha* strain Re2058/pCB113 was used for the production of poly(hydroxybutyrate‐*co*‐hydroxyhexanoate) P(HB‐*co*‐HHx) throughout this study (Budde et al., 2011). Strain Re2058 has a *proC* knockout and is consequently proline auxotrophic. The *proC* was inserted into the plasmid pCB113 for plasmid stability in antibiotic‐free mineral salt media (MSM). Strain Re2058 has a *phaC* deletion, but for PHA synthesis a *phaC* from *Rhodococcus aetherivorans* and *phaJ* from *Pseudomonas aeruginosa* are integrated on the plasmid pCB113.

The media compositions of tryptic soy broth (TSB), agar plates and MSM were described previously (Gutschmann et al., 2019). Preculture MSM contained 10 g L^−1^ of canola oil and 4.45 g L^−1^ of urea as the main carbon and nitrogen sources to adapt the culture to lipid consumption. The FPE was supplied from the company BIOIBERICA S.A.U. from their industrial heparin production process. MSM for shake flask screening and bioreactor cultivations contained the FPE as the sole carbon and nitrogen source. FPE was composed as previously reported: 53% dry matter, 38% fat with 49% free fatty acids, 11% total protein content and melting point of at least 100°C (Saad et al., 2021).

### Preculture cultivation conditions

A single colony from an agar plate, previously incubated for 3–4 days at 30°C, was used to inoculate the first preculture in 10 ml TSB media in a 125‐mL Ultra Yield flask (Thomson Instrument Company), sealed with an AirOtop membrane (Thomson Instrument Company). At an OD_600nm_ ≥5 after 13–15 h of incubation, 3 ml were transferred to a second preculture containing 300 ml MSM. The second preculture was incubated for 24 h in a 1000‐ml DURAN baffled glass flask (DWK Life Sciences GmbH) sealed with an AirOtop membrane. An orbital shaker (INFORS HT Multitron Standard, Infors AG) was used for the incubation of both precultures at 30°C, 200 rpm and with a shaking amplitude of 25 mm.

### Shake flask cultivations

The shake flask cultivations were conducted in a working volume of 50 ml MSM containing 5–50 g L^−1^ of the FPE as the sole carbon and nitrogen source. During media preparation, the FPE was directly weighted into each individual flask. After inoculation with 0.5 ml of the second preculture, the cultures were incubated for 72 h at 200 rpm at 30°C in a 250‐ml DURAN baffled glass flask sealed with an AirOtop membrane. The whole culture was harvested at the end of the cultivation for cell dry weight (CDW) determination. Cultivations with each feedstock concentration were performed in triplicate.

### Bioreactor cultivation conditions

The whole second preculture with a CDW of 1.35 ± 0.25 g L^−1^ was used to inoculate 2.7 L MSM containing 10 g L^−1^ of the FPE in a 6.6‐L laboratory bioreactor (BIOSTAT Aplus, Sartorius AG). The bioreactor was equipped with two six‐blade Rushton impellers with a distance of 96 mm. Due to heavy foaming, the MSM initially contained 3 ml antifoam agent (Nol‐LG126, Adeka). The cultures were aerated with air at a very low aeration rate of 0.5 L min^−1^ (0.167 vvm). The dissolved oxygen concentrations were maintained above 40% by an automated stirrer cascade between 200 and 600 rpm. 300 g of the FPE was fed to the bioreactor between 10 and 35 h by a newly developed feeding mechanism, which is described in the results section.

### Cell dry weight, polyhydroxyalkanoate and residual cell dry weight determination

For the determination of the CDW and PHA content during screening experiments, the whole shake flask culture was harvested in 50 ml polypropylene test tubes, whereas aliquots of 5–15 ml were sampled in 15‐ml polypropylene test tubes during bioreactor cultivations. The detailed procedures for gravimetric CDW determination by weighting and PHA determination by gas chromatography equipped with a flame ionization detector (GC‐FID) were described previously (Santolin et al., 2021). The residual cell dry weight (RCDW) was defined as CDW minus the PHA content in g L^−1^.

### Amino acid analysis

An aliquot of the supernatant (500 μl) was mixed with 500 μl of the internal standard (225 μM α‐amino‐butyric‐acid [ABA]). The mixture was washed twice with 1 ml hexane for 10 min in an overhead shaker to remove oleaginous residues. Hexane was removed and the washed supernatant was filtered through a 0.2 μm polytetrafluoroethylene (PTFE) syringe filter. Dilution series of a commercially available amino acid mixture (AAS18‐10x, Sigma‐Aldrich, Germany) were prepared for calibration purposes within a concentration range between 5 and 150 mg L^−1^. The amino acid concentrations were determined by high‐performance liquid chromatography with a fluorescence light detector (HPLC‐FLD 1200 series, Agilent Technologies) equipped with a reversed phase column (Gemini, 5 μm, 150 × 4.6 mm, 110 Å, Phenomenex) and a precolumn (Gemini, 5 μm, 30 × 4.6 mm, 110 Å, Phenomenex). The HPLC method was adapted from a previous study (Krömer et al., 2005): for derivatization, ortho‐phthalaldehyde (OPA) reagent and 9‐fluorenylmethyl chloroformate (FMOC) were used. Separation was achieved using 40 mM NaH_2_PO_4_ buffer as the polar phase and a 45:45:10 (v:v:v) mixture of acetonitrile:methanol:water as the non‐polar phase at a flow rate of 1 ml min^−1^ for 64 min. The columns were maintained at 40°C. OPA derivates were excited at 340 nm and detected at 450 nm and FMOC derivates were excited at 266 nm and detected at 305 nm.

## RESULTS AND DISCUSSION

To accelerate a transition towards a circular bioeconomy, novel opportunities of waste and by‐product stream valorization must be exploited. One of the key elements in circular bioeconomy is the constant availability of these waste streams for PHA production. In this study, we evaluated a by‐product stream from industrial‐scale heparin production as a low‐cost carbon and nitrogen source for PHA production, which is available throughout the year.

### Shake flask cultivations

**FIGURE 1:**
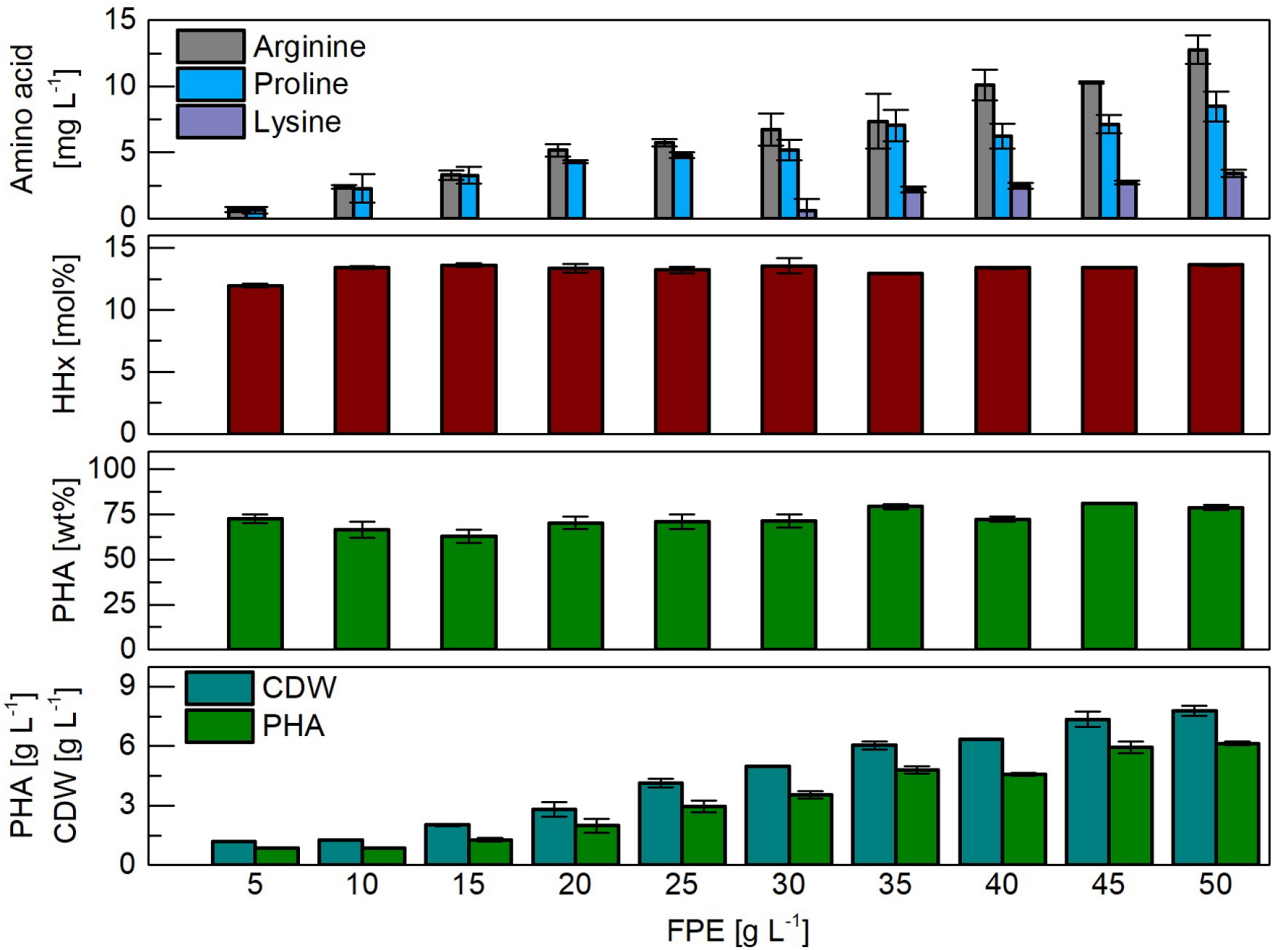
Shake flask cultivations of *R. eutropha* Re2058/pCB113 with a fat/protein‐emulsion (FPE) as sole carbon and nitrogen source. Cell dry weight (CDW, [g L^−1^]), polyhydroxyalkanoate (PHA, [g L^−1^]), PHA content per CDW [wt%], hydroxyhexanoate content per PHA (HHx, [mol%]) and amino acid concentration (mg L^−1^) after 72 h cultivation in mineral salt medium with different FPE concentrations (5–50 g L^−1^). Error bars indicate standard deviation from triplicate cultivations.

The impact of various concentrations of FPE as the sole source of nitrogen and carbon was examined firstly in shake flask cultivations (Figure 1). Irrespective of the FPE concentration, high amounts of PHA were accumulated. A maximum concentration of 81 wt% PHA was achieved while using 45 g L^−1^ FPE. Interestingly, with FPE concentrations between 10 and 50 g L^−1^, the hydroxyhexanoate (HHx) concentration stayed in between 12.9 and 13.6 mol% and did not significantly change. Nevertheless, an increasing CDW and PHA concentration was observed with a maximum of 7.8 g L^−1^ and 6.1 g L^−1^ respectively. These findings suggest that the C/N ratio is sufficiently high to induce nitrogen‐depleted conditions while carbon is in excess; otherwise, such a high intracellular PHA content would not be observable (Mitra et al., 2021). When the same feedstock was used at a concentration of 10 g L^−1^ in a previous study with urea as an additional nitrogen source, a higher CDW was obtained but the PHA content in the cells was lower (Saad et al., 2021). These findings suggest that the new media simplification is beneficial for achieving high intracellular PHA contents regardless of the FPE concentration, which is desirable for an economic PHA production process (Choi & Lee, 1999). Nevertheless, it might be beneficial to add a defined amount of urea per gram of FPE for an improved yield of active biomass. Interestingly, only three amino acids were detected in the supernatant at the end of the cultivation: arginine, lysine and proline (Figure 1). The lowest CDW were obtained when arginine and lysine were used as the sole source of carbon and nitrogen in another study (Kimura et al., 2003), which indicates that they are not a preferred substrate and also explains the obtained results in the present study.

Proline was among the amino acids that were detected in the supernatant (Figure 1). Although a proline auxotrophic strain was used for the cultivations and no kanamycin was added for plasmid maintenance, it can be concluded from the high PHA content that the plasmid pCB113 was maintained stable in the cells. Usually low‐copy number plasmids possess a special segregation system to ensure their segregation into the daughter cells (Ebersbach & Gerdes, 2005), but pCB113 does not have it (Budde et al., 2011). In our case, the *phaC* (PHA synthase gene) located on pCB113 might be an additional selection marker, as cells with the PHA accumulation capability under the nutrient limited conditions are more robust, which is a common physiological advantage among numerous bacteria compared to their PHA negative mutants (Obruca et al., 2020).

### Development of a pneumatic feeding system

To perform fed‐batch cultivations, a new system had to be developed to transfer the solid FPE into the bioreactor. The inability to liquefy the FPE made it impossible to pump the feedstock to the bioreactor as described in a previous study (Gutschmann, Maldonado Simões, et al., 2022).

**FIGURE 2:**
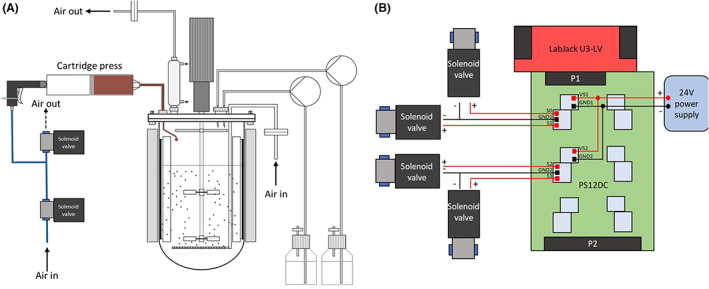
Set‐up of the pneumatic feeding system for solid fat/protein‐emulsions (FPEs). (A) Schematic of the bioreactor connected to a cartridge press, which is automatically triggered by solenoid valves: one builds up pressure to press the FPE into the bioreactor, the other releases the pressure to stop the feeding. (B) Wiring diagram to control two parallel cultivations.

The principal concept to transfer the substrate was to use high pressure for its delivery into the bioreactor. To realize it, a cartridge press was connected to the bioreactor and the press was pneumatically triggered using high‐speed solenoid valves (Figure 2A). The first valve was used to build pressure in the cartridge to press the substrate into the reactor, while the second valve was used to depressurize and thus stop the feed. The valve control was accomplished by a power switching board, which was controlled by a LabJack (LabJack Corporation) via LabView (National Instruments) (Figure 2B). The developed LabView routine allowed to set the opening times of the valves, the time between valve openings and number of pulses, which facilitated a tailored feeding profile. For instance, 0.3 g of the FPE could be added to a valve opening of 5000 ms at 1.5 bar. This set‐up allows the control of up to 12 valves in parallel, which means 6 bioreactors could be run in parallel. In this study, two bioreactor cultivations were performed in parallel as a test case with the shown valve wiring (Figure 2B). Additionally, a precision pressure reducing valve was installed at the air inlet to adapt the air pressure to the viscosity of the paste‐like substrate. In preliminary tests, the pressure reducing valve was not used, which led to rapid emptying of the cartridge due to excessive pressure. A detailed list of the applied and commercially available hardware used is shown in Table S1. A further important aspect was to maintain sterility. Therefore, the FPE was separately autoclaved in a beaker and transferred into the cartridge under a clean bench. Subsequently, a piston was inserted. Directly after autoclavation, the substrate became more flowable, which allowed to pour 300 g in a cartridge. Next, the cartridge was inserted into the cartridge press, which was then connected to the bioreactor and the pressurized air. The developed system should not only allow bioprocess development with the tested FPE, but it could also be used for other paste‐like substrates that are not pumpable.

### Bioreactor cultivations

**FIGURE 3:**
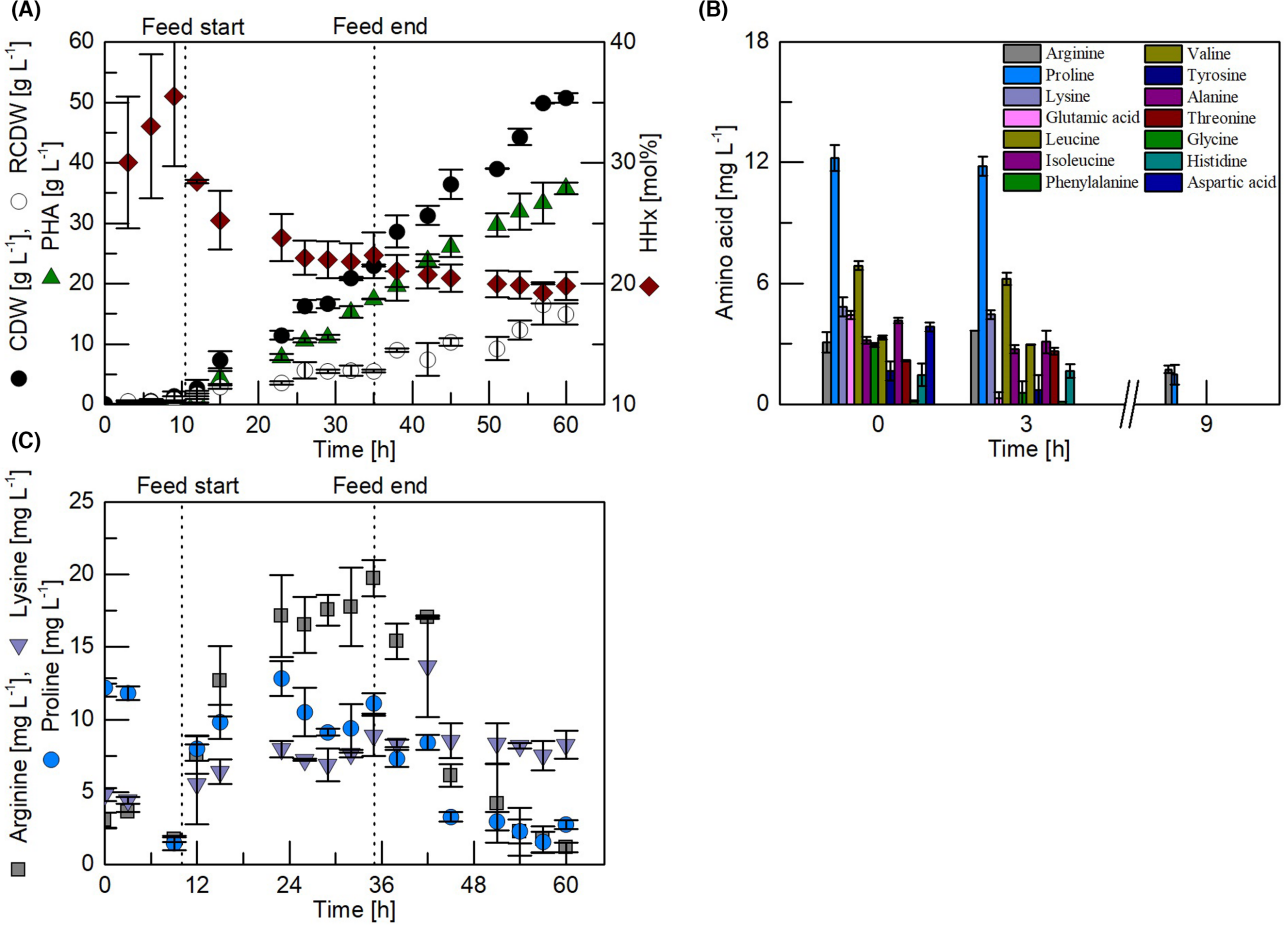
Fed‐batch cultivations for P(HB‐*co*‐HHx) production by *R*. *eutropha* Re2058/pCB113. The cultures initially contained 10 g L^−1^ of a solid fat/protein‐emulsion (FPE). A pneumatic feeding system was used to transfer 100 g L^−1^ of the solid FPE into the system from 10 to 35 h. (A) Cell dry weight (CDW) (g L^−1^), residual cell dry weight (RCDW) (g L^−1^), polyhydroxyalkanoate (PHA) (g L^−1^) and hydroxyhexanoate (HHx) content of PHA (mol%) are shown. (B) Amino acids detected at 0, 3 and 9 h of the process. (C) Arginine (mg L^−1^), lysine (mg L^−1^) and proline (mg L^−1^) profiles throughout the cultivation. All error bars show the maximum and minimum values of two replicate cultivations.

To evaluate the developed feeding system, two bioreactor cultivations were run in parallel using the FPE as the sole source of carbon and nitrogen. The CDW, RCDW and PHA increased until the end of the feeding phase to 23 g L^−1^ CDW containing 75 wt% PHA with a HHx content of 22.4 mol% and 5.5 g L^−1^ RCDW (Figure 3A). It is very likely that the cells encountered a substrate limitation during that phase, because a decrease in HHx from 10 h onwards was observed, which is a typical behaviour of this strain during nitrogen‐limiting conditions (Gutschmann et al., 2021; Riedel et al., 2012, 2015). A further increase in all parameters was observed until the end of the cultivation, which resulted in a final concentration of 35.8 g L^−1^ PHA with a HHx content of 19.8 mol%. This corresponds to an overall process yield of 0.60 g_PHA_ L^−1^ h^−1^. In total, a conversion of 0.33 g_PHA_ g_FPE_^−1^ was achieved. When neglecting the water content in the substrate of 47.2% (Saad et al., 2021), an even higher conversion yield of 0.70 g_PHA_ g_dry FPE_^−1^ was achieved. Such a yield is very similar to PHA yields reported for other cultivations with oleaginous (waste) feedstock (Lakshmanan et al., 2020; Riedel & Brigham, 2019, 2020).

A continuous single‐stage cultivation is an interesting approach for PHA production as it allows for overall higher space–time yields, a reduction of reactor downtime and a cost reduction associated with cleaning and sterilization (Koller, 2018). The observed simultaneous growth and PHA formation with a high intracellular PHA content could facilitate an implementation of such a continuous single‐stage process in the future, which is in contrast to low PHA contents with *R*. *eutropha* in a single‐stage chemostat reported in a different study (Zinn et al., 2003).

Small clumps of the substrate were visible in the media during the cultivation, which did not disintegrate directly, even though a fat emulsification by *R*. *eutropha* is well known (Lu et al., 2013; Riedel et al., 2014). Additionally, an increase of the RCDW to 15 g L^−1^ was observed until the end of the cultivation, which indicates a surplus of carbon and nitrogen sources throughout the process. While many amino acids were detected in the first 3 h of the process (Figure 3B), only asparagine, lysine and proline were detected from 9 h onwards (Figure 3C). It is important to emphasize that these three amino acids were present at a detectable concentration during the feeding phase, while other free amino acids were not. Since most of the amino acids were obviously taken up immediately, proline and arginine are not preferentially consumed. Not much have been published about the uptake of these two amino acids in *R*. *eutropha*. It seems, however, likely that their abundance throughout the cultivation is due to low uptake mechanisms. Arginine, in contrast to proline and lysine, seems not be taken up to a measurable extent throughout the fed‐batch phase at all. A limited nitrogen availability inside the cell is assumed when most of the other free amino acid concentrations declined after 9 h. This is probably the reason for a high PHA content (>70 wt%) after that time point, which also corresponds to the trend of the HHx content. Among the most abundant amino acids that were found in ^13^C‐labeling experiments after total hydrolysis in samples of *R*. *eutropha* were phenylalanine and tyrosine when grown on glycerol under aerobic conditions (Alagesan et al., 2018). It is likely that these amino acids are not only essential, but were incorporated into intracellular synthesis routes to a higher share. Therefore, correspondingly high uptake capacities of *R*. *eutropha* for these amino acids are likely. Glutamic acid, which is also not detectable in the fed‐batch phase, is commonly consumed at high rates in many bacteria.

In general, the presence of a complex nitrogen source in the FPE seemed beneficial for the developed process. Even though amino acids serve as a carbon and nitrogen source simultaneously, multiple studies report an advantage for PHA production by adding a defined amount of single amino acids or a complex nitrogen source to the media: The addition of only four single amino acids (methionine, histidine, leucine and arginine) lead to an improved growth and a shorter lag phase for *R*. *eutropha* (Azubuike et al., 2020). The PHA concentration could be increased from 3.6 g L^−1^ in the control cultivation to 7.5 g L^−1^ for yeast extract, 4.3 g L^−1^ for casein hydrolysate and 12.1 g L^−1^ for cheese whey hydrolysate in shake flask cultivations with *R*. *eutropha*, while the addition of soy peptone did not alter the PHB concentration, but increased the CDW by 1 g L^−1^ (Obruca et al., 2014). In the same study, bioreactor cultivations with cheese whey hydrolysate and a reduced ammonium sulphate content increased the space–time yield from 0.69 to 0.96 g_PHA_ L^−1^ h^−1^ (Obruca et al., 2014). In another study it was shown that the PHA concentration increased from 6.9 to 10.6 g L^−1^ when chicken feather hydrolysate was added as an inexpensive complex nitrogen source to *R*. *eutropha* cultures while the ammonium content was reduced simultaneously (Benesova et al., 2017). Lastly, it was also shown that *R*. *eutropha* can grow and synthesize PHA from single amino acids as the sole source of carbon and nitrogen (Kimura et al., 2003).

The developed process provides a suitable strategy for conversion of an animal by‐product stream to PHA, but cost‐effective PHA recovery remains a challenge. We showed that the bacteria efficiently take up the substrate, and an optimized C/N ratio will allow no residual lipids that potentially interfere with the downstream. Nevertheless, it is anticipated that the influence of residual lipids is neglectable when a non‐halogenated solvent‐based downstream processing is applied, which was shown for high‐purity P(HB‐*co*‐HHx) recovery from *R*. *eutropha* cells grown on plant oil and waste animal fat (Riedel et al., 2013; Thiele et al., 2021).

## CONCLUSION

In this study, we assessed the possibility of using FPE, a constantly available by‐product from industrial‐scale heparin production, as the sole source of carbon and nitrogen for PHA production. During scale‐up from shake flask to laboratory‐scale bioreactor cultivations, a feeding system was developed to perform fed‐batch cultivations with the solid FPE yielding 35.8 g L^−1^ P(HB‐*co*‐HHx) with a space–time yield of 0.6 g_PHA_ L^−1^ h^−1^. It can be concluded that such a feedstock is a very promising low‐cost substrate for PHA production. The demonstrated approach presents a novel option to valorize such animal by‐product streams.

## AUTHOR CONTRIBUTIONS

SLR and BG contributed to the conceptualization and design of the study. BG and THH constructed the pneumatic feeding system and realized the controller. BG, BH and MMS carried out the experiments. SJ supported the amino acid analysis quantification. BG, BH, and MMS carried out analysis of the data. BG prepared the first draft of the manuscript. SJ, TG, SLR and PN were responsible for the project administration and funding acquisition. All authors contributed to the manuscript revision and read and approved the submitted version.

## CONFLICT OF INTEREST

TG was employed by the company ANiMOX GmbH. All authors declare no conflict of interest.

## Data Availability Statement

The raw data supporting the conclusions of this article will be made available by the authors, without unduereservation.

## Supporting information


Table S1

## Endnote

^1^

www.bioiberica.com
