# Supplementary material for: Polyhydroxyalkanoate production from animal by‐products: Development of a pneumatic feeding system for solid fat/protein‐emulsions
Source: Microb Biotechnol. 2022 Sep 27;16(2):286–94. doi: 10.1111/1751-7915.14150 (PMC9871516; doi:10.1111/1751-7915.14150)
Supplement: Supplementary file 1 — Table S1 [file MBT2-16-286-s001.docx]

Supporting information

Polyhydroxyalkanoate production from animal by-products: Development of a pneumatic feeding system for solid fat/protein-emulsions

Björn Gutschmann^1^, Thomas H. Högl^1^, Boyang Huang^1^, Matilde Maldonado Simões^1^, Stefan Junne^1^, Peter Neubauer^1^, Thomas Grimm^2^, Sebastian L. Riedel^1,*^

^1^ Technische Universität Berlin, Chair of Bioprocess Engineering, Berlin, Germany

^2^ ANiMOX GmbH, Berlin, Germany

* Correspondence: [riedel@tu-berlin.de](mailto:riedel@tu-berlin.de)

Table S1. Commercially available parts that were used to build the pneumatic feeding system.

| Part | Company | Country |
| --- | --- | --- |
| Power switching board (PS12DC) | LabJack Corporation | USA |
| LabJack U3 (LV, HV) | LabJack Corporation | USA |
| Solenoid valve (MHJ10-S-2,5-QS-6-HF) | Festo SE & Co. KG | Germany |
| Manometer (MAP-40-4-1/8-EN) | Festo SE & Co. KG | Germany |
| Precision pressure reducing valve (MS6-LRP-1/2-D4-A8) | Festo SE & Co. KG | Germany |
| Cartridge press (DP310 PRO) | Aerotec | Germany |
| Cartridges (46.9 x 215 mm, 310mL, M15) | Tubex Wasungen GmbH | Germany |
| Harvest tube (0.5 mL, D_i_ = 4 mm, M10 x 1) | bbi-biotech GmbH | Germany |
